# Supplementary material for: Exploring ambivalence: A psychoanalytic analysis of emotional complexity in selected Amharic novels
Source: PLoS One. 2025 Jul 9;20(7):e0327631. doi: 10.1371/journal.pone.0327631 (PMC12240293; doi:10.1371/journal.pone.0327631)
Supplement: Attachment — Submitted filename: Response to Reviewers.docx [file pone.0327631.s003.docx]

**Rebuttal Letter**

Dear Editor ,

Thank you for your feedback and for the opportunity to revise and resubmit our manuscript titled “**Exploring Ambivalence: A Psychoanalytic Analysis of Emotional Complexity in Selected Amharic Novels**” (Manuscript ID: [PONE-D-24-51424). We greatly appreciate the time and effort of the reviewers and the academic editor in evaluating our manuscript. Based on their insightful comments, we have made extensive revisions to the manuscript, which we believe have significantly improved the quality and clarity of our work.

Please find below a detailed response to each of the reviewers' comments. We have addressed all points raised, making appropriate changes to the manuscript where necessary. For ease of reference, we have highlighted the revisions in the manuscript and included specific responses to each point.

We hope that the revisions meet the expectations of the reviewers and the editor. We are happy to make further changes if required and are looking forward to your feedback.

Thank you again for considering our manuscript.

Sincerely,

Dawit Dibekulu (PhD)

Reviewer response

PONE-D-24-51424

Title: **Exploring Ambivalence: A Psychoanalytic Analysis of Emotional Complexity in Selected Amharic Novels**

| No | Reviewer Comments | Author Response |
| --- | --- | --- |
| 1 | 1. Is the manuscript technically sound, and do the data support the conclusions?  The manuscript must describe a technically sound piece of scientific research with data that supports the conclusions. Experiments must have been conducted rigorously, with appropriate controls, replication, and sample sizes. The conclusions must be drawn appropriately based on the data presented.  Reviewer #1: Partly | I revised it and put in red coulor:  **Changes and Justifications:**   1. **Increased Data Support**: The revised version now incorporates specific references to the characters’ emotional and psychological struggles. For example, I added descriptions of key scenes from the novels (Semayneh’s hesitation in *Yäqënat Zār* and Besrat’s reflection in *Emba ɨnna Saq*), which demonstrate how the concepts of ambivalence are directly supported by textual evidence. These examples show how ambivalence influences character behavior and relationships. 2. **Methodological Rigor**: I explicitly tied the analysis to Freud’s model of the id, ego, and superego, clarifying how this theoretical framework was applied to understand the psychological dynamics of the characters. This demonstrates methodological rigor and connects your literary analysis to a well-known psychological theory. 3. **Contextualization**: The revised conclusion highlights how the emotional struggles of the characters are not just personal but also reflect broader social and historical contexts, such as Ethiopia’s historical tensions between tradition and modernity. This provides a clearer link between the micro (individual emotions) and macro (societal change) levels of analysis. 4. **Linking Conclusions to Evidence**: The emotional and psychological consequences of ambivalence are now shown to be based on specific textual data, ensuring that the conclusions are directly supported by the analysis of the novels. |
| 2 | Has the statistical analysis been performed appropriately and rigorously?  Reviewer #1: N/A | Since this study is a **literary analysis** of ambivalence in two contemporary Amharic novels, statistical methods were not applicable. Instead, the analysis was based on **thematic analysis** and **Freudian psychoanalysis**, using close reading techniques to identify and interpret key emotional and psychological conflicts in the characters. This method ensures that the conclusions are based on a rigorous and systematic interpretation of the texts." |
| 3 | Have the authors made all data underlying the findings in their manuscript fully available? The [PLOS Data policy](http://track.editorialmanager.com/CL0/http:%2F%2Fwww.plosone.org%2Fstatic%2Fpolicies.action%23sharing/1/010f019470d05cf4-d24246b4-b23b-4d83-959b-7e3e5524a919-000000/B7UhrpljgHWZj8hiX1CHfqv88oaqVgCOrwwDlNi7lYg=194) requires authors to make all data underlying the findings described in their manuscript fully available without restriction, with rare exception (please refer to the Data Availability Statement in the manuscript PDF file). The data should be provided as part of the manuscript or its supporting information, or deposited to a public repository. For example, in addition to summary statistics, the data points behind means, medians and variance measures should be available. If there are restrictions on publicly sharing data—e.g. participant privacy or use of data from a third party—those must be specified.  Reviewer #1: Yes | Yes all the data is available in the manuscript |
| 4 | Is the manuscript presented in an intelligible fashion and written in standard English? PLOS ONE does not copyedit accepted manuscripts, so the language in submitted articles must be clear, correct, and unambiguous. Any typographical or grammatical errors should be corrected at revision, so please note any specific errors here.  Reviewer #1: No | I tried to amend different typological errors |
| 5 | Review Comments to the Author Please use the space provided to explain your answers to the questions above. You may also include additional comments for the author, including concerns about dual publication, research ethics, or publication ethics. (Please upload your review as an attachment if it exceeds 20,000 characters)  Reviewer #1: I must commend author(s) for their attempts to examine multiplicity of identities across Amharic literature. It is pertinent given the transdisciplinarity and cross-cultural replicability concerning underrepresented Amharans. The indigenous knowledge, somewhat analyzed in the ms, provides certain depth to what it means reading Amharic literary works. However, some discussions are not so straightforward: | Thank you very much for your insightful comments and constructive feedback on the manuscript. I deeply appreciate your recognition of the attempt to examine the **multiplicity of identities** within Amharic literature. Indeed, one of the primary goals of this study is to explore how ambivalence, as portrayed in *Yäqënat Zār* and *Emba ɨnna Saq*, reflects broader societal themes, especially as they relate to the **psychological and emotional landscapes** of the characters and the cultural contexts they inhabit.  I am particularly grateful for your acknowledgment of the importance of **indigenous knowledge** in the analysis. The portrayal of emotions like ambivalence, mourning, and melancholy in Amharic literature offers a unique opportunity to delve into the specific cultural and psychological dimensions of **Amhara identity**. As you rightly pointed out, this study attempts to shed light on underrepresented perspectives, and I’m glad that this aspect resonated with you.  Regarding your comment that "some discussions are not so straightforward," I understand that certain aspects of the analysis may need more clarity or further elaboration. I will review those sections carefully to ensure that the connections between the characters' emotional struggles, cultural contexts, and theoretical frameworks are expressed more clearly. Specifically, I will aim to refine the explanations of **how ambivalence** and **psychological conflict** are represented and connected to **historical and societal themes**, as well as ensure that the analysis of **indigenous knowledge** is more explicitly tied to the broader discussion of the texts. I welcome any additional suggestions or guidance on which specific sections you felt required further refinement.  Once again, thank you for your constructive feedback, and I look forward to making the necessary revisions to improve the manuscript. |
| 1 | I wish the introduction had problematized the issue right away as opposed to seeing rehashed emphases of literary studies. This could be done by elaborating upon the 'sanitized' representation of Amharic literature across the wide-ranging studies of literature. That is, author(s) might better start with 1) problematization of issue and description of terms as used in ms, 2) thesis statement, 3) objective of paper, 4) significance of paper to PLOS ONE, and 5) review of literature. At the moment, the introduction does not seem to engage readers, belaboring on what it means to prioritize literature, which is a well-known argument already. | I tried to restructured andrewrite in the following way   1. Introduction 2. Statement of the problem 3. Objectives 4. Significances of the paper to PLOS ONE 5. Review of related literature |
| 2 | Strange to say, having the words "duality" and "identity" in title, author(s) make little to no effort in teasing out these key words which could have been a fascinating investigation. At the moment, author(s) dwell, in so many places in introduction, the centrality of literature and psychoanalytical domains that intersect with Amharic literature. | Thank you for your valuable feedback. I appreciate your comment about the use of the terms **“duality”** and **“identity”** in the title of the manuscript. You raise an important point regarding the expectation that these concepts be more explicitly explored throughout the study. I acknowledge that while these terms are central to the title, the manuscript could benefit from a deeper exploration of **identity** and **duality** as they relate to the characters’ emotional and psychological landscapes.  I agree that these terms hold significant potential for a **fascinating investigation**, and I appreciate that you have drawn attention to this. Moving forward, I will revise the manuscript to better unpack the **concepts of duality** and **identity**, especially in relation to the characters in *Yäqënat Zār* and *Emba ɨnna Saq*. Specifically, I will ensure that the analysis of the characters’ **internal conflicts**, **contradictory emotions**, and **psychological dilemmas** is framed within the context of **identity** and **duality**, emphasizing how these dynamics play out not only at the personal level but also in the broader societal and cultural contexts.  In addition, I will address the intersection of **literature** and **psychoanalysis** in a more integrated way, clearly illustrating how these theoretical frameworks illuminate the dualities present in the characters' experiences. By refining these connections, I aim to make the relationship between **identity**, **duality**, and **psychoanalysis** more explicit and more relevant to the overall themes of the manuscript.  Once again, thank you for your insightful comments. I am confident that these revisions will improve the manuscript’s depth and clarity, and I appreciate your thoughtful guidance. |
| 3 | Author(s) assume readers are well-aware of the intricate sociocultural, sociopolitical nuances of Amharans. While author(s) highlight the diverse tapestry of political, social, and cultural dimensions associated with Amharans, nowhere in introduction or review of literature section do author(s) 'inform' readers of specific sociocultural, sociopolitical 'confrontations' or problematics (was there indigenous community bloodshed? was there a largely, overlooked third gender? were there territorial disputes marginalizing the indigenous?), which could have been the key to unlocking the entire study. Readers ought to know the multiple ways in which Amharans as society play significant role to the publication of select texts. | hank you for your insightful feedback. I appreciate your comment about the **sociocultural** and **sociopolitical** context, and I understand the concern regarding the need to inform readers of the specific **sociopolitical confrontations** or challenges that might shape the psychological experiences of the characters. However, as this study is primarily focused on the **psychological** aspects of ambivalence in the selected novels, the emphasis remains on the **internal psychological states** of the characters and how their emotional and psychological struggles unfold within the narrative, rather than on broader **sociopolitical** or **historical** dynamics.  That said, I recognize that some readers may benefit from knowing the general **sociocultural** context in which the characters' psychological conflicts are embedded. In response, I will revise the **introduction** and **literature review** to clarify that while the analysis does not delve into **historical** or **sociopolitical** issues in detail, the **cultural backdrop** and **societal pressures** subtly influence the psychological struggles of the characters. This will include a brief mention of how **social expectations**, **family dynamics**, and **gender roles** might shape the characters' inner turmoil, without making these aspects the focus of the paper.  The primary aim of the manuscript is to explore **psychological ambivalence**—the emotional and psychological conflicts that arise within the characters due to their contradictory desires and unresolved dilemmas. I will ensure that the **sociocultural influences** on these psychological conflicts are clear but will refrain from making them the focal point of the study.  Thank you again for your thoughtful feedback, and I believe these revisions will clarify the scope of the study while maintaining the focus on the characters' psychological dynamics. |
| 4 | It goes without saying that literary studies employ, more often than not, textual analysis. Author(s) could have elaborated at length the importance of close reading of excerpts, but at the moment, author(s) seem to be fixated with notion of qualitative or otherwise, which does not seem to provide key bearings to how the ms is supposed to be positioned. Is this a literary or social science ms? If this is a literary work, close reading approaches should have been the key from which author(s) draw upon to introduce readers. At the moment, the research design needs a lot of work, particularly from literary studies' standpoint. | Thank you for your valuable feedback. I appreciate your comment regarding the need for a more explicit focus on **close reading** in the study, especially from a **literary studies** perspective. While the manuscript does incorporate theoretical frameworks and qualitative analysis, I agree that **close reading** of **textual excerpts** should be central to the analysis, particularly in a **literary study**. I will revise the manuscript to emphasize this approach, incorporating **detailed textual analysis** to better illustrate how the characters' psychological ambivalence is conveyed through specific passages. This will strengthen the research design and align the manuscript more clearly with **literary studies**. Thank you again for your constructive comments. |
| 5 | Currently, figure 1 has a lot of awkward spellings that could tarnish the journal's reputation. Please fix this. | I was fixing it. |
| 6 | While author(s) provide a section on framework, it seems that author(s) are interested merely in name dropping, listing who's who in psychoanalysis and its intersection of this in literature. Author(s) could have been more specific in the lenses used and introduced to readers particular theoretical/analytical configurations that are recontextualized to the investigation across Amharic literature. At the moment, author(s) do not tease out this, and assume readers are well-attuned to the specific operationalization of framework employed in the article. This needs to be revised precisely. | Thank you for your thoughtful feedback. I appreciate your comment regarding the theoretical and analytical framework. I understand the concern about the need for a more specific and contextually grounded explanation of how psychoanalytic theories are being applied to the analysis of the Amharic novels in this study.  In response to your feedback, I will revise the framework to clarify the **specific lenses** used and how these theoretical concepts are operationalized within the context of **Amharic literature**. Rather than simply listing the prominent figures in psychoanalysis, I will focus on how their theories—particularly Freud’s structural model of the psyche, Lacan’s emphasis on the Symbolic, and Klein’s object relations theory—are recontextualized to investigate the **psychological complexities** within the **selected novels**. The revised section will highlight how these psychoanalytic theories uniquely contribute to understanding the emotional and psychological landscapes of the characters in **Yäqënat Zār** and **Emba ɨnna Saq**, particularly in terms of **ambivalence.**  This revision will aim to ensure that readers understand how these frameworks are not only introduced but also **operationalized** to illuminate the psychological dimensions of the characters' experiences. I will also avoid assuming prior knowledge of these theories by providing a more detailed and clear explanation of how they are applied in this study. Thank you again for your constructive suggestions; I believe these changes will significantly improve the clarity and focus of the theoretical framework. |
| 7. | The notion of translated texts is interesting, but nothing has been said concerning verification of translation. Who verified what? What is academic qualification of these inter-rater experts? How many hours of translation training were received by these translator-experts? This information might better be considered by author(s). | I tried to mention in the methodology section translation approach |
| 8. | At the moment, analyses are not tied to the notion of "duality" and "identity," and the many ways in which these manifest in the grand scheme of psychoanalytical work involving said Amharic literature. Author(s) merely analyze without making scholarly and analytical links with the lenses as mentioned at the beginning of ms. | **The title is rephrase from “Navigating the Duality of Identity: A Psychoanalytic Exploration of Ambivalence in Selected Amharic Novels” to this**  **“Exploring Ambivalence: A Psychoanalytic Analysis of Emotional Complexity in Selected Amharic Novels” thus the issue is solved** |
|  | For these reasons, I recommended a major revision to the article. I wish author(s) the very best in revising said ms! | I revised the article thoroughly and marked all changes in red. This study is a psychoanalytic study focused on individual psychological identity, rather than on communal or cultural identity, which is internal. |
