# Supplementary material for: Exploring ambivalence: A psychoanalytic analysis of emotional complexity in selected Amharic novels
Source: PLoS One. 2025 Jul 9;20(7):e0327631. doi: 10.1371/journal.pone.0327631 (PMC12240293; doi:10.1371/journal.pone.0327631)
Supplement: Attachment — Submitted filename: Response_to_Reviewers_auresp_2.docx [file pone.0327631.s004.docx]

**Response to Reviewers**

Dear Editor and Reviewers,

Thank you for your constructive feedback on our manuscript, *"Exploring Ambivalence: A Psychoanalytic Analysis of Emotional Complexity in Selected Amharic Novels"* (PONE-D-24-51424R1). We appreciate the time and effort you have dedicated to reviewing our work. Below, we address each of the reviewers' comments and outline the changes made to the manuscript.

**Reviewer #1:**

1. **Comment:** The manuscript suffers from major sentence-level issues and requires substantial editing for readers' comprehension.
   **Response:** We have thoroughly revised the manuscript to improve clarity, grammar, and sentence structure. Key changes include:
   - Rewriting ambiguous or convoluted sentences for better readability.
   - Ensuring consistency in terminology and style.
   - Proofreading the entire document to eliminate typographical and grammatical errors.
2. **Comment:** The manuscript is partly technically sound.
   **Response:** We have strengthened the methodological rigor by:
   - Clarifying the theoretical framework and its application to the analysis.
   - Providing additional context for the selected novels to justify their inclusion.
   - Ensuring all conclusions are well-supported by the data presented.

**Reviewer #2:**

1. **Comment:** The manuscript shows clear improvements but needs further proofreading and minor restructuring, particularly in the introduction and theoretical sections.
   **Response:** We have:
   - Restructured the introduction to better frame the research question and its significance.
   - Streamlined the theoretical sections to enhance logical flow and coherence.
   - Conducted a detailed proofreading pass to address grammatical and language issues.
2. **Comment:** The study presents a strong and original exploration of ambivalence in Amharic literature.
   **Response:** We appreciate this acknowledgment and have further refined the discussion to highlight the originality and contributions of our work.

**Additional Actions Taken:**

- **Data Availability:** Confirmed that all underlying data are fully available and properly cited.
- **References:** Reviewed the reference list to ensure completeness and accuracy, including updates where necessary.
- **Figures:** Uploaded figures to the PLOS PACE tool to ensure compliance with journal requirements.

**Rebuttal Letter and Revised Files:**

- **Response to Reviewers:** This letter details all changes made in response to your comments.
- **Revised Manuscript with Track Changes:** Highlighted modifications for transparency.
- **Manuscript (Clean Version):** An unmarked copy of the revised manuscript.

We believe these revisions have significantly improved the manuscript and addressed all concerns raised. Thank you again for your valuable feedback. We look forward to your consideration of the revised version.

Sincerely,
Dawit Dibekulu (PhD)
